# Front-of-pack labeling and perceived nutritional quality in adults with and without chronic disease: results from a quasi-experimental study

**DOI:** 10.3389/fnut.2025.1736934

**Published:** 2026-02-09

**Authors:** Patricio Pérez-Armijo, Samuel Durán-Agüero, Enrique Echevarría-Orella, Luis Carlos Abecia-Inchaurregui, Unai A. Pérez de Arrilucea Le Floc’h, Rafael Almendra-Pegueros

**Affiliations:** 1 Department of Physiology, Faculty of Pharmacy, University of the Basque Country EHU, Vitoria-Gasteiz, Spain; 2 Faculty of Health Sciences, Universidad Isabel I, Burgos, Spain; 3 Facultad de Ciencias de la Rehabilitación y Calidad de Vida, Universidad San Sebastián, Santiago, Chile; 4 Department of Preventive Medicine and Public Health, Faculty of Pharmacy, University of the Basque Country EHU, Vitoria-Gasteiz, Spain; 5 Universidad Europea de Valencia, Faculty of Health Sciences, Nursing Department, Research Group Quality of Life and Health, Valencia, Spain; 6 Institut de Recerca Sant Pau (IR SANT PAU), Barcelona, Spain

**Keywords:** chronic disease - epidemiology, front-of-pack labeling (FOPL), Nutri-score, nutritional perception, warning labels

## Abstract

**Introduction:**

Noncommunicable diseases (NCDs) are a leading global health concern, linked to poor dietary choices and misperceptions about food healthfulness. Front-of-pack labeling (FOPL) systems, such as Nutri-Score (NS) and Warning Labels (WL), aim to guide healthier food choices, yet their effectiveness in populations with chronic conditions remains underexplored.

**Methods:**

We conducted a quasi-experimental online study among 5,140 adults in Spain, including individuals with and without NCDs. Participants evaluated the perceived nutritional quality of five commonly consumed foods under three different FOPL in the following order: no label (control), NS, and WL. The participants assessed each product in a randomized order within each label conditions to minimize order and learning effects.

**Results:**

Both labeling systems significantly influenced perceptions of nutritional quality (*p* < 0.001). NS tended to reduce the proportion of participants identifying products as low nutritional quality, particularly for items with a health halo such as yogurts and whole-wheat bread, while WL consistently increased the identification of products as low nutritional quality across all food types, including those traditionally perceived as healthy. This pattern was observed in both NCD and non-NCD groups.

**Discussion:**

While NS may inadvertently reinforce health halos for certain products, WL appear more effective in correcting misperceptions and promoting accurate assessments of nutritional quality in comparison to NS and no FOPL. These findings support the use of WL as a more impactful FOPL strategy for guiding informed food choices, particularly among individuals with chronic health conditions.

## 1 Introduction

Noncommunicable diseases (NCDs), including cardiovascular disease, type 2 diabetes, and obesity, are among the leading causes of global morbidity and mortality (1–3). A substantial share of their development is linked to unhealthy dietary patterns and to consumers’ difficulty recognizing the true nutritional quality of the products they choose (4, 5). In this context, front-of-pack labeling (FOPL) has been promoted by international organizations as a tool to improve understanding of nutrition information and support healthier purchase decisions (6, 7).

FOPL systems are typically classified as non-interpretive, which present numeric data without judging overall quality, and interpretive, which synthesize information into easily understood messages or symbols (6). Among the latter, Nutri-Score (NS) and Warning Labels (WL) stand out. NS assigns a color-letter grade from A (dark green) to E (red) based on the product’s overall nutritional profile (8). WL, first implemented in Chile, use black octagons with the statement “HIGH IN” to alert consumers when thresholds for sugar, saturated fat, sodium, or calories are exceeded (9).

FOPL effectiveness depends on several factors, including design, comprehension, and the ability to shape perceptions (10). The latter is especially relevant for foods that carry a “health halo,” a socially or commercially constructed positive image that leads consumers to overestimate nutritional value. Such halos may stem from ingredients, nutrient or health claims, or brand prestige, and are used in marketing to increase consumption of lower-quality products (11). This phenomenon distorts perceived healthfulness and encourages underestimation of energy content, reducing adherence to dietary recommendations (12).

Within this context, evidence suggests that NS can prompt consumers to purchase products graded A or B, visually associated with green hues (13). Yet, in many cases, these products may still exceed one or more critical nutrients, generating a health halo and encouraging more favorable perceptions than their composition warrants (14, 15). Research also shows that consumers often interpret green as signaling wellness or naturalness, even when products are high in sugar, fat, or sodium (16, 17). By contrast, WL operate through a different mechanism: their high-contrast design and direct wording trigger an immediate alert and shift perception, helping consumers identify products as less healthy (10, 18, 19).

Most available studies, however, focus on the general population (18, 20). There is limited evidence on how FOPL affects people living with chronic disease, who may have different motivations and dietary needs. Spain, marked by a strong Mediterranean food culture and the coexistence of different labeling formats on the market, offers a particularly relevant setting to examine how FOPL type can reshape perceived nutritional quality. For that reason, we aimed to compare changes in perceived nutritional quality of foods commonly viewed as healthy under NS versus WL among adults in Spain with and without chronic disease, to assess the potential of each system to correct health halos and guide more informed food choices.

## 2 Materials and methods

### 2.1 Study design

We conducted a quasi-experimental repeated-measures study with a cross-sectional structure, administered online via a self-completed questionnaire. All participants were sequentially exposed to three FOPL conditions in the following order: (a) no label (control), (b) NS, and (c) WL. Each participant served as their own control, allowing direct comparison of changes in perceived nutritional quality across systems. Image order was randomized within each label conditions to minimize order and learning effects. The STROBE guidelines for observational studies were follow (21).

### 2.2 Population, sample recruitment and ethics aspects

Eligible participants were adults of any sex residing in Spain with internet access. We included respondents who self-reported either living with diet-related NCDs or without such conditions, and who provided their electronic informed consent. People with severe visual impairments that could interfere with the visual task and incomplete questionnaires were not included.

The minimum sample size was set at 385 participants with NCDs, based on the Spanish European Health Survey (2020), which indicates a prevalence of 54.3% of adults living with chronic diseases in Spain (22, 23), with a confidence of 95, and 5% margin of error. The non-NCD group was recruited through non-probability convenience sampling with snowball distribution to reach a similar size. Participants were recruited through the networks of national patient associations, which helped disseminate the study to their members, and via public calls on social media, email, and health- and nutrition-related digital platforms. Participation was voluntary, anonymous, and without compensation. Data collection occurred during the first quarter of 2021.

The research was conducted in accordance with the Declaration of Helsinki of 1975 and its subsequent amendments. The study was approved by the Ethics Committee for Research Involving Human Participants of the University of the Basque Country (CEISH, Ref. M10/2020/053MR4) and classified as minimal risk.

### 2.3 Instrument design and validation

#### 2.3.1 Foods selected and front-of-pack labeling conditions

**Figure 1:**
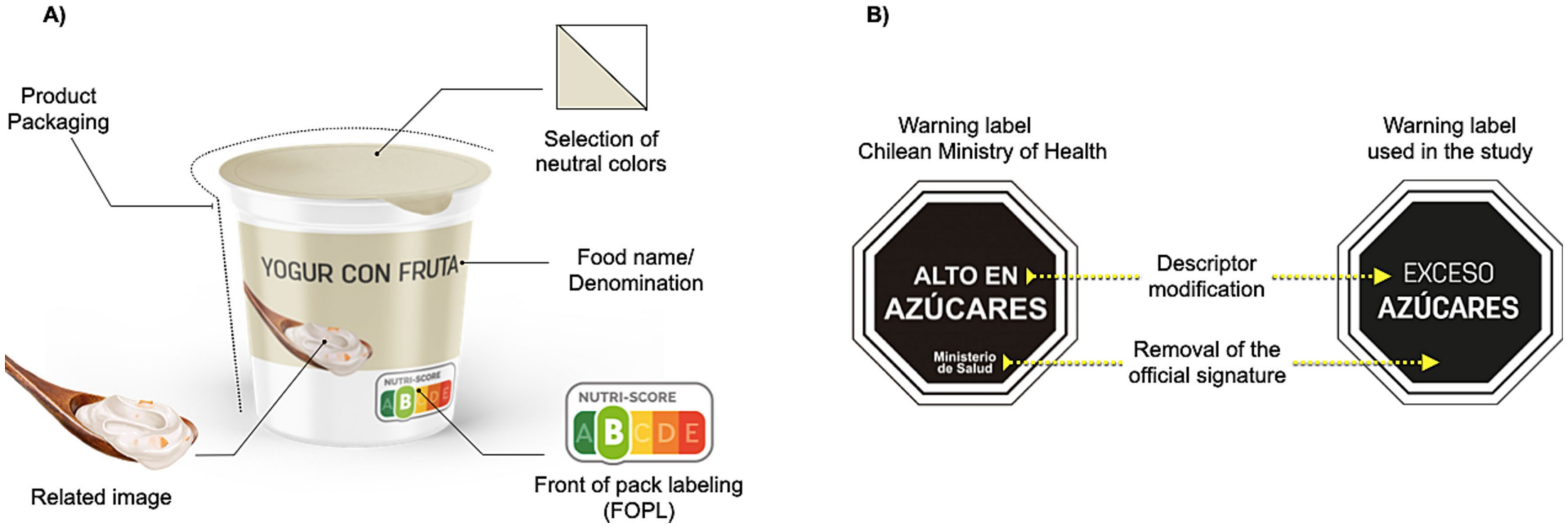
Graphic design and Warning Label (WL) adaptation for food products used in the study. **(A)** Technical and conceptual elements used in the design of the study products. In this example, a yogurt product was used. **(B)** WL used in this study. In this example, only show the WL for sugar content.

We selected five commonly consumed foods socially perceived as healthy (Greek yogurt, fruit yogurt, corn–flake–type breakfast cereal, sliced whole-wheat bread, and orange juice), given their association with everyday eating, particularly breakfast (24). Study images were custom-designed without brands or promotional elements, using neutral packaging and uniform backgrounds (Figure 1A). FOPL appeared at the lower-right corner of the pack and was repeatedly enlarged beneath the image to aid readability.

For the calculation of the NS, the original model, prior to the 2022 modification, was used, which employs a five-category A–E gradient of overall nutritional quality (8). Also, all the products tested were identified using online listings from Spanish supermarket chains with broad assortments that had begun voluntary NS application to private-label items (Carrefour Spain; Eroski), supplemented by the Carrefour France catalogue, where NS was more widespread at that time. For the WL condition, the recommendations and thresholds for sugar, saturated fat, sodium, and calories from Chile’s Ministry of Health (MINSAL) were followed, and a black octagon bearing “EXCESS OF” was used instead of the “HIGH IN” descriptor. This change was made to avoid potential confusion, since under European regulations the term “HIGH IN” is considered a nutrition claim (25). In addition, to improve label visibility and minimize distracting elements, the lower section of the warning labels (signature or brand reference) indicating the “Ministry of Health” was removed. Consequently, the final descriptors used were “EXCESS” of “SUGARS,” “SATURATED FAT,” “SODIUM,” and “CALORIES,” when the corresponding nutrient exceeded the threshold (9, 26). Per MINSAL limits, all selected foods exceeded at least one critical nutrient (Figure 1B). In Supplementary Table S1, the complete nutritional composition of the selected products is listed.

#### 2.3.2 Sociodemographic and health data

In the instrument designed, a section for sociodemographic and health data was added to collect information on sex, age, place of residency, educational level, employment status, economic income, and the presence or diagnosis of non-communicable chronic diseases (NCDs).

#### 2.3.3 Instrument validation

The final version of the instrument was incorporated into a questionnaire in *Google Forms*; it comprised two relevant sections: (a) sociodemographic and health data; (b) an experimental task on perceived nutritional quality by the presence or absence of the FOPL. The content validity of the instrument was assessed using the Lawshe method with 21 experts in nutrition and health sciences (Supplementary Table S2), who evaluated the adequacy of each item, which integrates the complete questionnaire. The global Content Validity Index (CVI) of this instrument was 0.91, which indicates an adequate instrument for research, and the specific CVI for the dimension *“Assessment of the ability to interpret nutritional quality,”* where the data in this manuscript come from, was 1.0. (Supplementary Table S3). A pilot test with 30 participants confirmed the clarity of instructions and the image rendering.

### 2.4 Procedure

**Figure 2:**
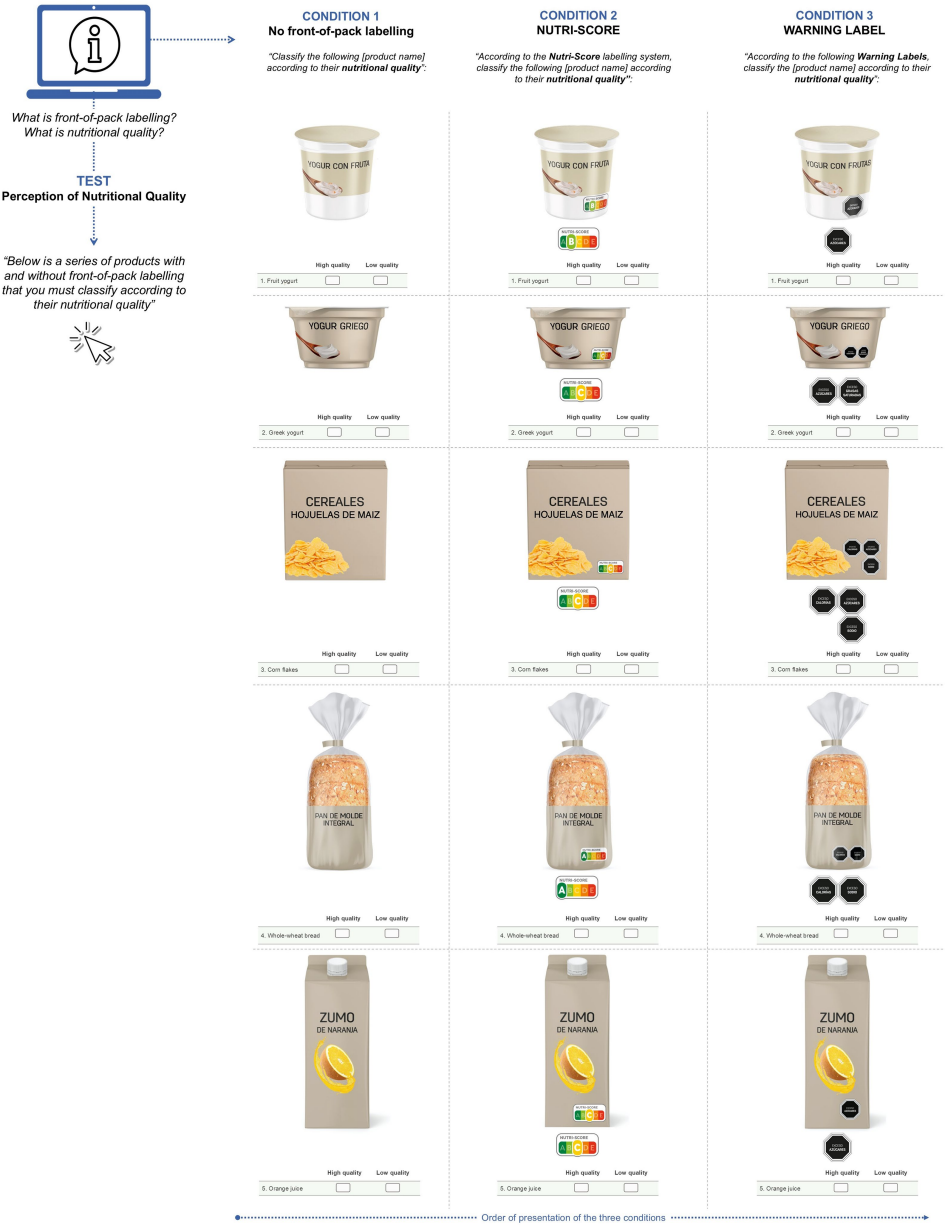
Elements and graphic representation of the five products based on three study conditions: without front-of-pack labeling (control), Nutri-Score, and warning labels.

After participants provided their electronic informed consent, information on sociodemographic and health data was retrieved. Details on *“nutritional quality”* and how to interpret FOPL (which you can see at the Supplementary Table S4) were given in written and graphic information. Each participant viewed all three FOPL conditions in the following order: Control, NS, and WL; this order was established to reduce potential order or familiarity bias and to prevent the warning format, which directly alerts consumers when a product exceeds critical nutrient thresholds, from influencing participants’ later perception of the products presented with NS (Figure 2). The five food items were presented in a randomized way, and at the start of each FOPL condition, participants were reminded about the presence or absence of FOPL and the task flow. In each FOPL condition, the participants must classify each product as “high” or “low nutritional quality.”

### 2.5 Statistical analysis

A descriptive statistical analysis was performed, and all the variables were presented in a dichotomous or categorical way, with absolute frequency and proportion. For the inferential statistical analysis, the Cochran’s Q test following by the McNemar and Pairwise comparisons using the Wilcoxon sign test as a *post hoc* analysis with a False Discovery Rate (FDR) correction was performed on the perceived nutritional quality of each product as “low nutritional quality” in each FOPL condition, this test was run separately for the NCD and non-NCD groups. All the analyses were conhducted in R software version 4.4.3 and RStudio 2025.09.1 (R Foundation for Statistical Computing, Vienna, Austria. https://www.r-project.org/foundation/), considering as statistically significant a *p* value of <0.05.

## 3 Results

### 3.1 Participants’ characteristics

We analyzed 5,140 valid responses; 23 questionnaires were excluded (incomplete or without consent). The sample was mainly female (75.4%) and 18–39 years (66.7%). Nearly half (46.1%) reported a university degree, and most (89.9%) reported being wholly or partly responsible for household food shopping. Overall, 38.2% reported living with an NCD, being the most common overweight/ or obesity, cardiovascular disease, or type 2 diabetes. The interest in food and nutrition was high (92.6%), though only 59.6% felt they had sufficient knowledge on food and nutrition (Table 1).

**Table 1:** Participant characteristics: demographics, health status, and nutrition-related behaviors.

Variable	*n* = 5,140% (n)
Sex	
Female	75.4 (3,878)
Male	24.6 (1,262)
Age (years)	
18–29	34.7 (1,783)
30–39	32.0 (1,647)
40–49	22.1 (1,136)
50–59	8.2 (422)
≥ 60	3.0 (152)
Education	
None	0.1 (6)
Primary/Secondary	22.3 (1,148)
Higher	46.1 (2,372)
Postgraduate	31.4 (1,614)
Chronic disease (any)	38.2 (1,966)
Overweight/Obesity	78.3 (1,540)
Cardiovascular disease	15.3 (301)
Type 2 diabetes	6.4 (125)
Member of patient association	5.1 (261)
Interest in food/nutrition	
None	0.3 (17)
Low	7.1 (365)
Quite interested	56.1 (2,884)
Very interested	36.5 (1,874)
Primary food shopper	
Yes	49.8 (2,563)
No	9.1 (467)
Shared equally	41.1 (2,110)

### 3.2 FOPL and perceived nutritional quality in people living with chronic disease

**Figure 3:**
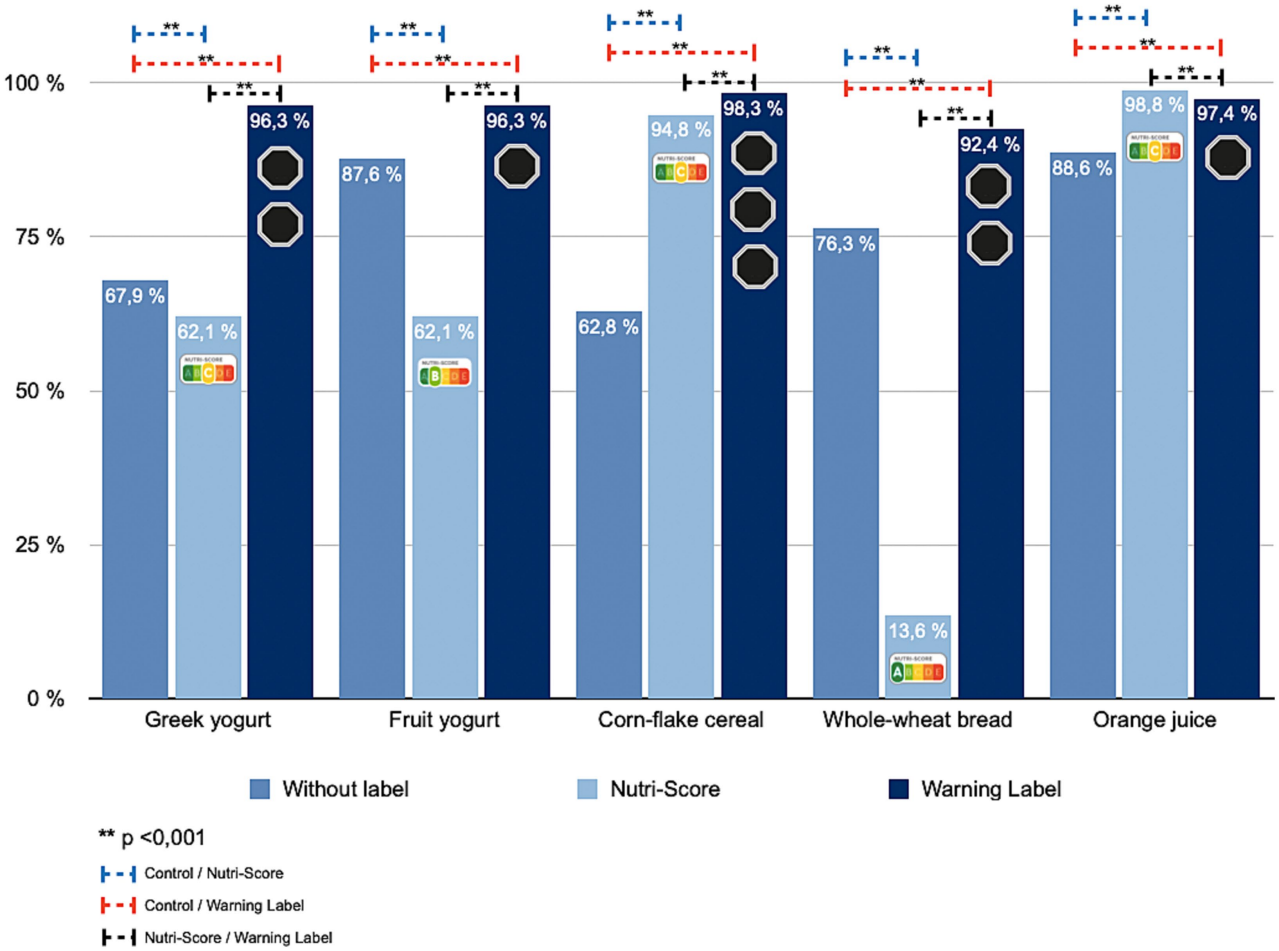
Percentage change in the identification of unhealthy products according to the front-of-pack labeling displayed among participants with chronic disease.

Among respondents with NCDs, perceived nutritional quality varied significantly across FOPL conditions (*p* < 0.001). For Greek yogurt and fruit yogurt, 67.9% initially judged the products as low quality. Under NS, that share fell to 62.1%, but under WL it rose sharply to 96.3% (Table 2; Figure 3). Corn-flake–type cereal showed increased low-quality identification with both labels, more pronounced for WL (*p* < 0.001). For whole-wheat sliced bread, ratings moved inversely: 76.3% low quality at baseline, 13.6% under NS, and 92.4% under WL. For orange juice, 88.6% rated it low quality without labels, 98.8% under NS, and 97.4% under WL (*p* < 0.001).

**Table 2:** Change in identification of products as “unhealthy” across FOPL conditions (control, NS, WL) in participants with chronic disease (Cochran’s Q; *p*-values).

Selected food products	Population with chronic disease			
	Without FOPL % (*n*)	Nutri-Score % (*n*)	Warning Labels % (*n*)	*p*-value Cochran’s Q test
Greek yogurt	67.9 (1,335)	62.1 (1,221)	96.3 (1,893)	<0.001
Fruit yogurt	87.6 (1,723)	62.1 (1,221)	96.3 (1,893)	<0.001
Corn-flake cereal	62.8 (1,235)	94.8 (1,864)	98.3 (1,933)	<0.001
Whole-wheat bread	76.3 (1,501)	13.6 (268)	92.4 (1,817)	<0.001
Orange juice	88.6 (1,742)	98.8 (1,942)	97.4 (1,914)	<0.001

### 3.3 FOPL and perceived nutritional quality in people without chronic disease

**Figure 4:**
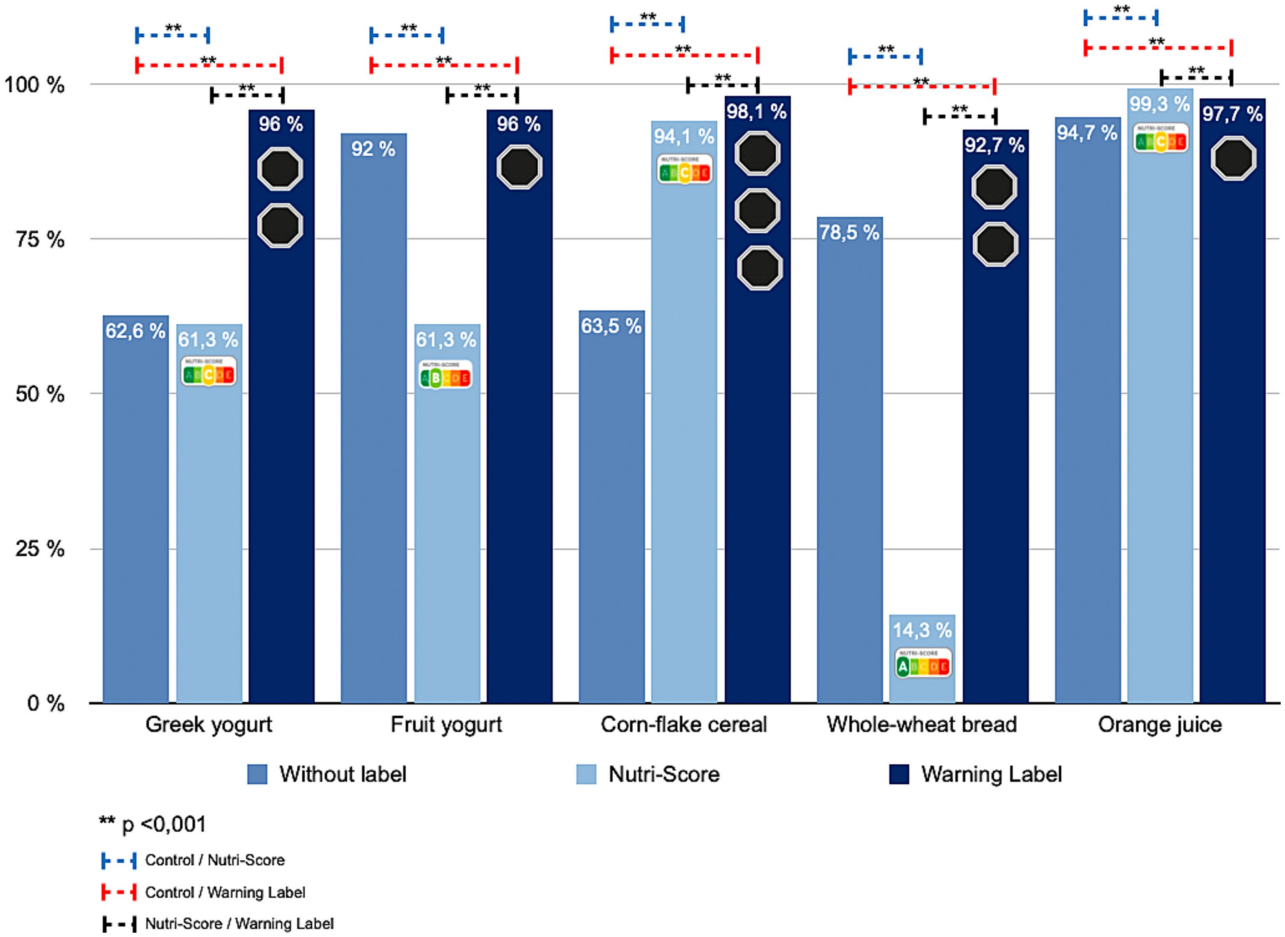
Percentage change in the identification of unhealthy products according to the front-of-pack labeling displayed among participants without chronic disease.

Findings were similar in participants without NCDs (*p* < 0.001) (Table 3; Figure 4). For Greek yogurt, 62.6% rated it low quality without labels, 61.3% under NS, and 96.0% under WL. Fruit yogurt showed a comparable pattern (slight decrease with NS to 61.3%, increase to 96.0% with WL). For corn-flake cereal, low-quality identification rose in both labeling conditions, more strongly for WL (*p* < 0.001). Whole-wheat bread saw a marked decline under NS (14.3%) versus 78.5% no label and 92.7% under WL (*p* < 0.001). For orange juice, low-quality perception was highest under NS, followed by WL (*p* < 0.001).

**Table 3:** Change in identification of products as “unhealthy” across FOPL conditions (control, NS, WL) in participants without chronic disease (Cochran’s Q; *p*-values).

Selected food products	Population without chronic disease			
	Without FOPL % (*n*)	Nutri-Score % (*n*)	Warning Labels % (*n*)	*p*-value Cochran’s Q test
Greek yogurt	62.6 (1,988)	61.3 (1,946)	96.0 (3,047)	<0.001
Fruit yogurt	92.0 (2,921)	61.3 (1,946)	96.0 (3,047)	<0.001
Corn-flake cereal	63.5 (2,017)	94.1 (2,988)	98.1 (3,113)	<0.001
Whole-wheat bread	78.5 (2,493)	14.3 (453)	92.7 (2,943)	<0.001
Orange juice	94.7 (3,007)	99.3 (3,151)	97.7 (3,102)	<0.001

## 4 Discussions

The increase in the prevalence of NCDs in recent years (2, 3) and their impact on health systems worldwide have fostered the development and implementation of public policies to improve the quality of diets, such as the adoption of FOPL (7). Along with this, research that will lead us to better FOPL systems is still needed. For example, their effectiveness varies depending on the format and on how each system influences consumers’ perception of nutritional quality (13). Understanding these differences is crucial to assessing real-world usefulness. For that reason, this study aimed to compare changes in perceived nutritional quality of foods commonly viewed as healthy under two different label conditions: NS and WL, in adults living with and without chronic disease in Spain. To our knowledge, this is one of the first studies aimed at identifying the role of FOPL in the perception of nutritional quality of foods in people living with NCDs.

The main results showed that WL consistently increased the identification of products as low nutritional quality across all food types studied, including those traditionally perceived as healthy. Across both groups, a similar pattern emerged; the NS shows a reduction of negative perceptions for some food products, whereas the WL increases the perception of lower nutritional quality for the same set of products. These findings are similar to those reported by Ares et al. (10), where FOPL, like NS and health-star rating, increased the healthful perception of products, although these differences were product-dependent. These results underscore the need to implement a system that objectively enables consumers to select foods of higher nutritional quality.

In the case of Greek yogurt, fruit yogurt, and whole-wheat bread, we observed a reduction in the proportion of people with and without NCDs who classified these products in the category of low nutritional quality when they were shown with the NS label condition. This observation could reflect their placement within the A or B NS categories, displayed in green hues. According to Schuldt, green has been shown to signal healthfulness, especially to consumers engaged with healthy eating (17). Along with this, previous works reported that NS labels only affect the perception of nutritional quality of products in the red or green extremes categories, just like happens in our research (16, 27). Moreover, the impact of NS label colors could impact the purchase intentions, increasing the purchase of products in green categories, without affecting the purchase of those in the red category (27). This finding is particularly relevant given reports indicating that approximately 40% of products classified in categories A and B correspond to ultra-processed foods according to the NOVA classification, which have been associated with poorer health outcomes (15, 28). Additionally, in the case of fruit yogurt may also benefit from a socially reinforced “healthy” image due to fruit content combined with green labeling (29).

In contrast, when these products were shown with the WL condition, the perception of low nutritional quality increased; this observation aligns with the system’s threshold-based nature: labels appear only when critical nutrients exceed cutoffs, creating a “discovery effect” that clarifies which products are less healthy (30). Qualitative studies in Chile have reported positive consumer appraisals on the benefits of WL to clarify the lack of healthfulness of certain food products that had traditionally been marketed as healthy; this was observed across all the socioeconomic strata (30).

These findings are consistent with Centurión et al., and with Arrúa et al. (31, 32), who found that perceptions of product healthfulness were only significantly affected by nutritional warnings, which were associated with less healthy products compared to those that did not display such warnings (31); and also, this effect is higher in comparison to other FOPL such as Guideline Daily Amount (GDA), and Multiple Traffic Light (MTL) (32). In addition, other studies report that changes in the perception of the nutritional quality of products with warning labels go beyond reducing purchase intention, as they also increase motivation to buy products with better nutritional quality (33), that could impact in the reformulation of products (34).

The case of corn-flake–type cereal reinforces this pattern: most participants rated it as low quality when three WL were present (“HIGH IN calories,” “HIGH IN sugars,” “HIGH IN sodium”), suggesting a possible cumulative effect. However, we could not analyze the impact of the number or type of label on product perception in this study. Prior work by Machín et al. (35) found that fat warnings elicited more negative perceptions than sugar or sodium warnings, highlighting the need to consider the nutrient signaled when evaluating consumer response. In addition, Crovetto et al. have reported that after 5 months of WL in Chile, more than 60% of the consumers stopped choosing a food item because of the number of labels on the packaging (36).

On the other hand, the orange juice, a product traditionally perceived as healthy, had low-quality ratings that were slightly higher under NS (category C) in comparison to WL. However, we did not assess whether these slight differences corresponded to statistically significant differences, although it is clear that both FOPL conditions have an important influence on reducing the perceived nutritional quality of this product (37, 38). The modest change here may reflect the presence of a single warning (“HIGH IN sugars”), interpreted as a moderate risk (35).

While NS may inadvertently reinforce health halos for certain products, WL appear more effective in correcting misperceptions and promoting accurate assessments of nutritional quality. These findings support the use of WL as a more impactful FOPL strategy for guiding informed food choices, particularly among individuals with chronic health conditions. However, more studies are needed to confirm our results and test other hypotheses, like the effect of the number of WL, packaging, marketing, preferences, beliefs about food, income, and other factors on the nutritional perception of food and the purchase intention.

### 4.1 Limitations and strengths

This study presents some limitations, one of which is the predominant participation of females and young, with a high educational level and a strong interest in food and nutrition. This pattern is consistent with previous research showing greater participation of women, younger adults, and individuals with higher education and income in online nutrition surveys (39) and could be explained by the easier digital access in comparison to older adults (40). On the other hand, in terms of research, the order in which the FOPL conditions were presented was fixed and may have resulted in order or carryover effects. However, the sequence was selected to reduce the potential impact of warning labels on the assessment of Nutri-Score; but these effects cannot be entirely ruled out and should be considered when interpreting the results. Also, the results are based on a virtual test after receiving educational information that may increase nutrition awareness in comparison to the one observed during the real purchase phenomenon, where the loyalty to brands, nutrition/health claims and the full nutrition panel could influence the nutritional quality perceived of the selected food (41), for that reason, more studies addressing the influences of these factors is still needing. However, along with the use of a properly validated instrument, the main strength of this study was the large sample size of participants with and without NCDs to perform the comparison analysis. Finally, the statistical and methodological procedures ensure the accuracy and validity of the results by providing convincing evidence.

## 5 Conclusion

In conclusion, exposure to either NS or WL FOPL alters the perceived nutritional quality in comparison to the control condition in the groups studied. NS showed a tendency to create a health halo for products categorized as higher quality especially those signaled in green (A/B) even when some exceeded critical nutrients (e.g., fruit yogurt, Greek yogurt, whole-wheat bread). WL, in turn, increased low-quality perceptions across all tested products, including those with a socially healthy image, supporting their capacity to correct misperceptions and promote more appropriate choices among adults with and without NCDs.

## Data Availability

The raw data supporting the conclusions of this article will be made available by the authors, without undue reservation.

## References

[1] Taheri Soodejani M. Non-communicable diseases in the world over the past century: a secondary data analysis. Front Public Health. (2024) 12:1436236. doi: 10.3389/fpubh.2024.1436236, 39421825 PMC11484412

[2] World Health Organization. Noncommunicable diseases (2025). Available online at: https://www.who.int/news-room/fact-sheets/detail/noncommunicable-diseases [Accessed October 30, 2025]

[3] Li J Pandian V Davidson PM Song Y Chen N Fong DYT. Burden and attributable risk factors of non-communicable diseases and subtypes in 204 countries and territories, 1990-2021: a systematic analysis for the global burden of disease study 2021. Int J Surg. (2025) 111:2385–97. doi: 10.1097/JS9.0000000000002260, 39869379 PMC12372739

[4] UN-Nutrition: the United Nations inter-agency coordination mechanism for nutrition. Non-communicable diseases, diets and nutrition. (2018). Available online at: https://www.unnutrition.org/library/briefs/non-communicable-diseases-diets-and-nutrition [Accessed October 30, 2025]

[5] Zhu M Xu S Li Y Wang W Liu L Xu Q . Global burden of non-communicable diseases attributable to behavioral factors. Sci Bull. (2025) 70:3129–33. doi: 10.1016/j.scib.2025.08.037, 40887373

[6] Kelly B Jewell J In: WHO Regional Office for Europe, editor. What is the evidence on the policy specifications, development processes and effectiveness of existing front-of-pack food labelling policies in the WHO European Region? Copenhagen: (2018) 30484994

[7] Hyseni L Atkinson M Bromley H Orton L Lloyd-Williams F McGill R . The effects of policy actions to improve population dietary patterns and prevent diet-related non-communicable diseases: scoping review. Eur J Clin Nutr. (2017) 71:694–711. doi: 10.1038/ejcn.2016.23, 27901036 PMC5470099

[8] Chantal J Hercberg S. Development of a new front-of-pack nutrition label in France: the five-colour Nutri-score. Public Health Panor. (2017) 3:712–25.

[9] Reyes M Garmendia ML Olivares S Aqueveque C Zacarías I Corvalán C. Development of the Chilean front-of-package food warning label. BMC Public Health. (2019) 19:906. doi: 10.1186/s12889-019-7118-1, 31286910 PMC6615240

[10] Ares G Varela F Machin L Antúnez L Giménez A Curutchet MR . Comparative performance of three interpretative front-of-pack nutrition labelling schemes: insights for policy making. Food Qual Prefer. (2018) 68:215–25. doi: 10.1016/j.foodqual.2018.03.007

[11] Peloza J Ye C Montford WJ. When companies do good, are their products good for you? How corporate social responsibility creates a health halo. J Public Policy Mark. (2015) 34:19–31. doi: 10.1509/jppm.13.037

[12] Provencher V Jacob R. Impact of perceived healthiness of food on food choices and intake. Curr Obes Rep. (2016) 5:65–71. doi: 10.1007/s13679-016-0192-0, 26820622

[13] Song J Brown MK Tan M MacGregor GA Webster J Campbell NRC . Impact of color-coded and warning nutrition labelling schemes: a systematic review and network meta-analysis. PLoS Med. (2021) 18:e1003765. doi: 10.1371/journal.pmed.1003765, 34610024 PMC8491916

[14] Romero Ferreiro C Lora Pablos D Gómez de la Cámara A. Two dimensions of nutritional value: Nutri-score and NOVA. Nutrients. (2021) 13:2783. doi: 10.3390/nu13082783, 34444941 PMC8399905

[15] Ebner P Frank K Christodoulou A Davidou S. How are the processing and nutrient dimensions of foods interconnected? An issue of hierarchy based on three different food scores. Int J Food Sci Nutr. (2022) 73:770–85. doi: 10.1080/09637486.2022.2060951, 35403522

[16] Bossuyt S Custers K Tummers J Verbeyst L Oben B. Nutri-score and nutrition facts panel through the eyes of the consumer: correct healthfulness estimations depend on transparent labels, fixation duration, and product equivocality. Nutrients. (2021) 13:2915. doi: 10.3390/nu13092915, 34578792 PMC8467654

[17] Schuldt JP. Does green mean healthy? Nutrition label color affects perceptions of healthfulness. Health Commun. (2013) 28:814–21. doi: 10.1080/10410236.2012.725270, 23444895

[18] Mediano Stoltze F Busey E Taillie LS Dillman Carpentier FR. Impact of warning labels on reducing health halo effects of nutrient content claims on breakfast cereal packages: a mixed-measures experiment. Appetite. (2021) 163:105229. doi: 10.1016/j.appet.2021.105229, 33789168

[19] Franco-Arellano B Vanderlee L Ahmed M Oh A L’Abbé M. Influence of front-of-pack labelling and regulated nutrition claims on consumers’ perceptions of product healthfulness and purchase intentions: a randomized controlled trial. Appetite. (2020) 149:104629. doi: 10.1016/j.appet.2020.104629 32061707

[20] Jürkenbeck K Mehlhose C Zühlsdorf A. The influence of the Nutri-score on the perceived healthiness of foods labelled with a nutrition claim of sugar. PLoS One. (2022) 17:e0272220. doi: 10.1371/journal.pone.0272220, 35976882 PMC9385015

[21] von Elm E Altman DG Egger M Pocock SJ Gøtzsche PC Vandenbroucke JP. Strengthening the reporting of observational studies in epidemiology (STROBE) statement: guidelines for reporting observational studies. BMJ. (2007) 335:806–8. doi: 10.1136/bmj.39335.541782.AD, 17947786 PMC2034723

[22] Instituto Nacional de Estadística. Encuesta europea de salud en España. INEbase. (2020). Available online at: https://www.ine.es/dyngs/INEbase/es/operacion.htm?c=Estadistica_C&cid=1254736176784&menu=resultados&idp=1254735573175 [Accessed December 15, 2025]

[23] Plataforma de Organizaciones de Pacientes. Observatorio de la atención al paciente. Informe_ (2021). Available online at: https://www.plataformadepacientes.org/sites/default/files/informe2021_oap_vf_2.pdf? [Accessed December 15, 2025]

[24] Ballco P Gracia A. Tackling nutritional and health claims to disentangle their effects on consumer food choices and behaviour: a systematic review. Food Qual Prefer. (2022) 101:104634. doi: 10.1016/j.foodqual.2022.104634

[25] Regulation (EC) No 1924/2006 of the European Parliament and of the Council of 20 December 2006 on nutrition and health claims made on foods. (2006). Available online at: http://data.europa.eu/eli/reg/2006/1924/oj [Accessed December 28, 2025]

[26] Ministerio de Salud de Chile. Reglamento Sanitario de los Alimentos. (2024). Available online at: https://www.minsal.cl/wp-content/uploads/2015/10/DECRETO_977_96_actualizado_-mayo-2024.pdf [Accessed December 15, 2025]

[27] De Temmerman J Heeremans E Slabbinck H Vermeir I. The impact of the Nutri-score nutrition label on perceived healthiness and purchase intentions. Appetite. (2021) 157:104995. doi: 10.1016/j.appet.2020.104995, 33068665

[28] Srour B Fezeu LK Kesse-Guyot E Allès B Méjean C Andrianasolo RM . Ultra-processed food intake and risk of cardiovascular disease: prospective cohort study (NutriNet-santé). BMJ. (2019) 365:l1451. doi: 10.1136/bmj.l1451, 31142457 PMC6538975

[29] Wąsowicz G Styśko-Kunkowska M Grunert KG. The meaning of colours in nutrition labelling in the context of expert and consumer criteria of evaluating food product healthfulness. J Health Psychol. (2015) 20:907–20. doi: 10.1177/1359105315580251, 26032806

[30] Ikonen I Sotgiu F Aydinli A Verlegh PWJ. Consumer effects of front-of-package nutrition labeling: an interdisciplinary meta-analysis. J of the Acad Mark Sci. (2020) 48:360–83. doi: 10.1007/s11747-019-00663-9

[31] Centurión M Machín L Ares G. Relative impact of nutritional warnings and other label features on cereal Bar healthfulness evaluations. J Nutr Educ Behav. (2019) 51:850–6. doi: 10.1016/j.jneb.2019.01.021, 30819654

[32] Arrúa A Machín L Curutchet MR Martínez J Antúnez L Alcaire F . Warnings as a directive front-of-pack nutrition labelling scheme: comparison with the guideline daily amount and traffic-light systems. Public Health Nutr. (2017) 20:2308–17. doi: 10.1017/S1368980017000866, 28625228 PMC10262271

[33] Adasme-Berríos C Aliaga-Ortega L Schnettler B Parada M Andaur Y Carreño C . Effect of warning labels on consumer motivation and intention to avoid consuming processed foods. Nutrients. (2022) 14:1547. doi: 10.3390/nu14081547, 35458109 PMC9029137

[34] Garcia VR Dieste PVG. Cambios en la presencia de edulcorantes no nutritivos en alimentos y bebidas dulces post implementación de la Ley de Promoción de la Alimentación Saludable en la ciudad de Buenos Aires, Argentina. Rev Esp Nutr Hum Diet. (2024) 28:220–31. doi: 10.14306/renhyd.28.3.2186

[35] Machín L Aschemann-Witzel J Curutchet MR Giménez A Ares G. Traffic light system can increase healthfulness perception: implications for policy making. J Nutr Educ Behav. (2018) 50:668–74. doi: 10.1016/j.jneb.2018.03.005, 29627330

[36] Crovetto M Acosta M Rocco Y. Ley 20.606: Efectos en el conocimiento de etiquetado nutricional en consumidores de un supermercado en Valparaíso de Chile: estudio descriptivo, cuanticualitativo, antes y después de 5 meses de la implementación de la ley. Rev Esp Nutr Hum Diet. (2020) 24:311–23. doi: 10.14306/renhyd.24.4.979

[37] Hock K Acton RB Jáuregui A Vanderlee L White CM Hammond D. Experimental study of front-of-package nutrition labels’ efficacy on perceived healthfulness of sugar-sweetened beverages among youth in six countries. Prev Med Rep. (2021) 24:101577. doi: 10.1016/j.pmedr.2021.101577, 34976639 PMC8683942

[38] Jáuregui A White CM Vanderlee L Hall MG Contreras-Manzano A Nieto C . Impact of front-of-pack labels on the perceived healthfulness of a sweetened fruit drink: a randomised experiment in five countries. Public Health Nutr. (2022) 25:1094–104. doi: 10.1017/S1368980021004535, 34726144 PMC9991717

[39] D’Ancona Ma ÁC. Survey quality in digital society: advances and setbacks. Rev Esp Investig Sociol. (2025) 4:25–42. doi: 10.5477/cis/reis.191.25-42

[40] Ministerio de Asuntos Económicos y Transformación Digital. Informe de cobertura de banda ancha en España en el año 2021. 2022. Available online at: https://avance.digital.gob.es/banda-ancha/cobertura/Paginas/Informe-de-cobertura-2021.aspx [Accessed October 31, 2025]

[41] Dumitru I Gârdan DA Paștiu CA Muntean AC Gârdan IP. On the mechanism of the label perception: how does labeling change food products customer behavior? Econ Comput Econ Cybern Stud Res. (2021) 55:193. doi: 10.24818/18423264/55.2.21.12

