# Supplementary material for: Front-of-pack labeling and perceived nutritional quality in adults with and without chronic disease: results from a quasi-experimental study
Source: Front Nutr. 2026 Feb 9;12:1736934. doi: 10.3389/fnut.2025.1736934 (PMC12936437; doi:10.3389/fnut.2025.1736934)
Supplement: Supplementary file 1 [file Table_1.pdf]

Supplementary Material

Table S1. Nutritional values of the selected products per 100 g or 100 ml

**Table S1.** Nutritional values of the selected products per 100 g or 100 ml						
**Product**	**Energy**		**Saturated fat** g/100 g	**Total sugars** g/100 g	**Salt** g/100 g	**Sodium** mg/100 g
	kJ/100 g	kcal/100 g				
Fruit yogurt	377	90	1.3	13	0.12	48
Greek yogurt	533	128	4.9	12	0.09	36
Corn-flake cereal	1,588	375	0.3	15	1	400
Whole-wheat bread	1,264	302	1.2	3.7	1.34	536
**Product**	**Energy**		**Saturated fat** g/100 ml	**Total sugars** g/100 ml	**Salt** g/100 ml	**Sodium** mg/100 ml
	kJ/100 ml	kcal/100 ml				
Orange juice	182	43	0	8.9	0	0

Table S2. Experts' profiles for the content validation analysis in the instrument design and validation process.

Table S2.
Experts' profiles for the content validation analysis in the instrument design and validation process.

Expert	Academic Background	Highest Academic Degree	Education and Training	Years of Experience
1	Environmental Sciences	PhD	PhD in Biological Sciences; Bachelor's degree in Environmental Sciences	35
2	Sociology	PhD	PhD in Sociology; Bachelor's degree in Sociology	31
3	Pharmacy	PhD	PhD in Pharmacy; Bachelor's degree in Pharmacy	31
4	Human Nutrition and Dietetics	PhD	PhD in Medicine; Master's degree in Clinical Nutrition; Bachelor's degree in Human Nutrition and Dietetics	26
5	Human Nutrition and Dietetics	PhD	PhD in Nutrition; Master's degree in Human Nutrition and Metabolism; Bachelor's degree in Human Nutrition and Dietetics	24
6	Human Nutrition and Dietetics; Food Science and Technology	PhD	PhD in Nutrition; Master's degree in Gastronomic Management and Innovation and Food Sciences; Master's degree in Nutrition Sciences; Bachelor's degree in Human Nutrition and Dietetics	18
7	Human Nutrition and Dietetics	PhD	PhD in Health Sciences; Master's degree in Public Health; Master's degree in Human Nutrition and Food Quality; Master's degree in Hospital and Social Services Management; Bachelor's degree in Human Nutrition and Dietetics	13
8	Pharmacy	PhD	PhD in Pharmacy; Bachelor's degree in Pharmacy	13
9	Nursing	PhD	PhD in Public Health; Master's degree in Public Health; Bachelor's degree in Nursing	7
10	Human Nutrition and Dietetics; Food Science and Technology	Master's	Bachelor's degree in Human Nutrition and Dietetics; Bachelor's degree in Food Science and Technology	26
11	Human Nutrition and Dietetics; Food Science and Technology	Master's	Master's degree in Hospital and Social Services Management; Bachelor's degree in Human Nutrition and Dietetics; Bachelor's degree in Food Science and Technology	16
12	Human Nutrition and Dietetics; Food Science and Technology	Master's	Bachelor's degree in Human Nutrition and Dietetics; Bachelor's degree in Food Science and Technology; Master's degree in Food Quality and Safety	11
13	Human Nutrition and Dietetics; Food Science and Technology	Master's	Bachelor's degree in Human Nutrition and Dietetics; Bachelor's degree in Food Science and Technology	11
14	Human Nutrition and Dietetics	Master's	Master's degree in Human Nutrition and Food Quality; Bachelor's degree in Human Nutrition and Dietetics	19
15	Human Nutrition and Dietetics	Master's	Master's degree in nutrition and Metabolism; Master's degree in Kinanthropometry and Sports Nutrition; Bachelor's degree in Human Nutrition and Dietetics	16

16	Human Nutrition and Dietetics	Master's	Master's degree in nutrition and food quality; Master's degree in Pharmacology, Nutrition and Sports Supplementation; Bachelor's degree in Human Nutrition and Dietetics	16
17	Human Nutrition and Dietetics	Master's	Master's degree in marketing and advertising communication; Bachelor's degree in Human Nutrition and Dietetics	16
18	Human Nutrition and Dietetics	Master's	Master's degree in Business Management and Marketing; Master's degree in Dietetics and Nutrition; Bachelor's degree in Human Nutrition and Dietetics	15
19	Human Nutrition and Dietetics	Master's	Bachelor's degree in Human Nutrition and Dietetics; Bachelor's degree in Food Science and Technology	11
20	Human Nutrition and Dietetics	Master's	Master's degree in Clinical and Community Nutrition; Bachelor's degree in Human Nutrition and Dietetics	4
21	Human Nutrition and Dietetics	Master's	Postgraduate training in Nutritional Coaching and new approaches to patient care; Bachelor's degree in Human Nutrition and Dietetics	4

The study included a panel of 21 professionals from diverse fields related to health, nutrition, food safety, sociology, and food education, ensuring a broad and multidisciplinary approach to the object of analysis.

Participants demonstrated strong academic backgrounds, with undergraduate degrees in Human Nutrition and Dietetics, Food Science and Technology, Pharmacy, Nursing, Sociology, and Environmental Sciences. Regarding academic level, nine experts held a doctoral degree, while the remaining nine had completed master's or specialized postgraduate training.

The panel members' professional experience ranged from 4 to 35 years and covered areas such as clinical and community nutrition, public health, food safety, research, healthcare management, as well as communication and education in nutrition.

1 **Table S3. Content Validity Ratio (CVR) of the entire instrument and its corresponding dimensions.**

Table S3.
Content Validity Ratio (CVR) of the entire instrument and its corresponding dimensions.

Dimension	Item	Item description	CVR
**Prior knowledge of nutrition and food**	1	Are you concerned about issues related to food and nutrition?	0.52
	2	What is your level of knowledge about nutrition?	0.71
	3	Which of the following diseases do you think are associated with a diet high in sodium, sugar, fat, and/or calories?	1.00
**Attitude toward nutrition information**	4	Are you responsible for food purchasing in your household?	1.00
	5	How important is nutrition information in your purchasing decision?	0.90
	6	How often do you consult nutrition information?	1.00
	7	When consulting nutrition information, to what extent do you find it easy to identify healthier products?	0.90
	8	Which nutrition information do you usually consult?	1.00
	9	For what reasons do you not consult nutrition information?	1.00
	10	For what reasons do you consult nutrition information?	1.00
	11	How important is the price of food in your purchasing decision?	0.90
**Perceived usefulness of the Nutri-Score system**	12	From the following scale, indicate the colors you consider most positive	1.00
	13	How useful do you consider the proposal to include Nutri-Score labeling to convey nutrition information?	0.71
	14	Do you think Nutri-Score labeling would help you make healthier purchasing decisions?	1.00
	15	Would you trust a product that does not carry Nutri-Score labeling?	0.81
	16	Would you replace a product you usually consume with another one that has a healthier Nutri-Score label?	1,00
	17	If a product you usually consume changes its flavor and/or texture (less sugar, fat, and/or salt) to make it healthier, would you buy it?	1.00
	18	Do you consider Nutri-Score labeling to be one of the measures that could help reduce obesity rates in the Spanish population?	0.90
	19	Do you consider Nutri-Score labeling to be one of the measures that could help reduce the risk of developing diet-related diseases such as obesity, hypertension, diabetes, cardiovascular disease, and some types of cancer?	0.81
	20	In your opinion, which of these two front-of-pack food labels would help you better identify the nutritional quality of a product?	1.00
**Assessment of the ability to interpret**	21	Products without front-of-pack labeling: classify the following foods according to their nutritional quality	1.00
	22	Products with Nutri-Score labeling: based on the following Nutri-Score labels, classify the following foods according to their nutritional quality	1.00

**nutritional quality**	23	Products with warning labels: based on the following warning symbols, classify the following foods according to their nutritional quality	1.00
**Assessment of changes in purchase intention**	24	Products without front-of-pack labeling: which of these foods would you buy?	0.81
	25	Products with Nutri-Score labeling: based on the following Nutri-Score labels, which of these foods would you buy?	0.90
	26	Products with warning labels: based on the following warning symbols, which of these foods would you buy?	0.90
**Content Validity Index (CVI)**			**0.91**

2 Table S4. Text on nutritional quality and front-of-pack labelling for consumers' educational purposes.

Table S4. Text on nutritional quality and front-of-pack labelling for consumers' educational purposes.

Term	Description	Reference
**Nutritional quality**	"Nutritional quality depends on the nutrient content of foods. Those that provide significant amounts of various nutrients are considered high quality, while those that provide only calories, contain excess sugars, saturated fats, and sodium (salt), are considered low"	(1)
**Nutri-Score**	"To facilitate the understanding of mandatory nutritional information, the Nutri-Score labelling system will be implemented in Spain as a voluntary front-of-pack logo displayed on the main face of food packages. Nutri-Score provides consumers with an overall assessment of the nutritional quality of a product based on its content of nutrients and food components to be encouraged—such as fiber, protein, fruits, vegetables, legumes, nuts, rapeseed oil, walnut oil, and olive oil—and nutrients to be limited, including energy, saturated fat, sugars, and salt. Each product is positioned on a five-level scale ranging from the most nutritionally favorable option (classified as A) to the least favorable option (classified as E). The corresponding category is highlighted in the logo by a larger letter"	(2)
**Warning Labels**	"Other countries use different front-of-pack labelling systems, including warning labels indicating "EXCESS." These labels consist of black octagons with white lettering that identify whether a food product exceeds established thresholds for sugars, saturated fat, sodium, and/or total energy. This system allows consumers to easily identify, at a glance, less healthy products and to preferentially choose foods of higher nutritional quality, either without warning labels or with fewer warning labels"	(3)

(1) Gobierno de Uruguay, Ministerio de Desarrollo Social. Guía alimentaria para la población uruguaya. 2019. Available on: https://www.gub.uy/ministerio-desarrollo-social/comunicacion/publicaciones/guia-alimentaria-para-la-poblacion-uruguaya

(2) Chantal J, Hercberg S, Development of a new front-of-pack nutrition label in France: the five colour Nutri-Score. Public Health Panorama. (2017) 03 (04):712-25.

(3) Reyes M, Garmendia ML, Olivares S, Aqueveque C, Zacarías I, Corvalán C. Development of the Chilean front-of-package food warning label. BMC Public Health. (2019) 19:906. doi: 10.1186/s12889-019-7118-1
